# Prevalence and risk factors for youth suicidality among perinatally infected youths living with HIV/AIDS in Uganda: the CHAKA study

**DOI:** 10.1186/s13034-020-00348-0

**Published:** 2020-10-24

**Authors:** Godfrey Zari Rukundo, Richard Stephen Mpango, Wilber Ssembajjwe, Kenneth D. Gadow, Vikram Patel, Eugene Kinyanda

**Affiliations:** 1 Department of Psychiatry, Mbarara University of Science and Technology, Mbarara, Uganda; 2 MRC/UVRI and LSHTM Uganda Research Unit, Mental Health Project, Entebbe, Uganda; 3 Statistical Section, MRC/UVRI and LSHTM Uganda Research Unit, Entebbe, Uganda; 4 Department of Psychiatry, Stony Brook University, Stony Brook, New York, USA; 5 Department of Global Health and Social Medicine, Harvard Medical School, Massachusetts, USA

**Keywords:** Suicide, Suicidality, Suicidal attempt, Suicidal ideation, Youths, HIV

## Abstract

**Background:**

Research from high income countries indicates that suicide is a major mental health care concern and a leading cause of preventable deaths among children and adolescents. Proper assessment and management of youth suicidality is crucial in suicide prevention, but little is known about its prevalence and associated risk factors in Sub-Saharan Africa. In low income countries there is an increased risk of suicide among persons with HIV/AIDS even in the presence of the highly active antiretroviral therapy.

**Objective:**

To determine the prevalence of and risk factors for youth suicidality among perinatally infected youth living with HIV/AIDS in Uganda.

**Methods:**

We studied 392 HIV positive children (5–11 years) and adolescents (12–17 years) and their caregivers in Kampala and Masaka districts. Caregivers were administered the suicide assessment section of the MINI International Psychiatric Interview. Socio-demographic characteristics, psychiatric diagnoses, and psychosocial and clinical factors were assessed and suicidality (suicidal ideation and or suicidal attempt) was the outcome variable. Logistic regression was used to calculate odds ratios adjusting for study site and sex at 95% confidence intervals.

**Results:**

Caregivers reported a suicidality rate of 10.7% (CI 8–14.1) in the past one month with higher rates among urban female (12.4%, CI 8.6–17.7) than male (8.7%, CI 5.4–13.8) youth. Lifetime prevalence of attempted suicide was 2.3% (n = 9, CI 1.2–4.4) with the highest rates among urban female youth. Among children, caregivers reported a lifetime prevalence of attempted suicide of 1.5%. The self-reported rate of attempted suicide in the past month was 1.8% (n = 7, CI 0.8–3.7) with lifetime prevalence of 2.8% (n = 11, CI 1.6–5.0). The most common methods used during suicide attempts were cutting, taking overdose of HIV medications, use of organophosphates, hanging, stabbing and self-starvation. Clinical correlates of suicidality were low socioeconomic status (OR = 2.27, CI 1.06–4.87, p = 0.04), HIV felt stigma (OR = 2.10, CI 1.04–3.00, p = 0.02), and major depressive disorder (OR = 1.80, CI 0.48–2.10, p = 0.04). Attention-deficit/hyperactivity disorder was protective against suicidality (OR = 0.41, CI 0.18–0.92, p = 0.04).

**Conclusion:**

The one-month prevalence of suicidality among CA-HIV was 10.7%.

## Background

Suicide is a major mental health care concern and a leading cause of preventable deaths among children and adolescents although there is some evidence that global rates may be going down [1–4]. There is limited data on youth suicide in developing countries especially sub-Saharan Africa, but it is generally agreed that its correlates are similar to middle to high income countries [5]. Among the most commonly reported clinical correlates are being female; bullying; exposure to violence; alcohol and drug use; mental disorders; and weak family structure to include physical and sexual abuse and poor interpersonal relationships [6–8]. Many studies report higher suicidality among females but higher completed suicides among male [3]. Suicidal ideation and attempts are major risk factors for future completed suicide. Proper assessment and management of suicidal ideation and attempts are crucial in suicide prevention. The burden and clinical correlates for suicide among children and adolescents in Sub-Saharan Africa (SSA) are not well known [3].

Previous research indicates increased risk of suicide among youth living with HIV even for individuals receiving highly active antiretroviral therapy (HAART) [9–11]. The relationship between HIV and suicide is commonly conceptualized as psychological pathway through perceived stigma, depression, hopelessness, low self-esteem, unemployment, socioeconomic hardships, low social support, and suicidal ideation, coupled together with predisposition [12, 13]. These variables appear to be similar in both youth and adults [14]. In addition to the psychosocial factors, individuals with HIV also experience challenges with clinical symptoms that subjects them to additional difficulties [15]. The greatest HIV/AIDS burden is born by sub-Saharan Africa (SSA), a region with the youngest populations [16, 17]. Suicidality is often comorbid with many physical and psychological conditions that are commonly not assessed or attended to. Youth with HIV find it difficult to disclose their suicidality due to the associated stigma and cultural unacceptability.

This paper describes the prevalence and the correlates of comorbid suicidality among youths living with HIV/AIDS in Kampala and Masaka districts of Uganda. We adopted the broad description of suicidality, a term that has been used in different ways to refer to suicidal ideation, suicidal behaviors, suicidal attempts and completed suicide [18, 19]. Our findings may be beneficial to the youths with suicidality, families and caregivers, healthcare professionals, scientists, and policy makers.

## Methods

### Participants and procedures

The study looked at 392 adolescents between the age of 12–17 years and their caregivers. We also interviewed caregivers of children aged 5–11 years. All the participants were from 5 study clinics located in Kampala and Masaka districts (Lubowa Joint Clinical Research Centre, Nsambya homecare department Children’s HIV Care clinic; Nsambya hospital, the Children’s clinic at The AIDS Support Organisation; TASO Masaka, Uganda Cares/Masaka Regional Referral Hospital and Kitovu Mobile AIDS Organisation, Masaka). Both adolescents with HIV and caregivers had to speak English or Luganda (the local language spoken in the study areas). Exclusion criteria were concurrent enrolment in other studies, need of immediate medical attention, and inability to understand the study’s assessment instruments. We administered the suicide assessment section of the MINI International Psychiatric Interview. Suicidality was defined as having suicidal attempt in the past or having suicidal ideation.

### Measures

Suicidality was assessed using the Mini-International Neuropsychiatric Interview (MINI) section on suicidality [20]. The MINI-KID is a structured diagnostic interview that is used to diagnose psychiatric disorders. We asked the caregivers the following questions: (S1) In the past month, did this child deliberately injure him/herself? (S2) In the lifetime of this child has he/she ever deliberately injured him/herself? (S3) If yes to S1 or S2, which method(s) of self-injury has this child used in the past?

The adolescents were asked various questions as indicated here below. In the past month, did you think about hurting or injuring yourself or have mental images of harming yourself, with at least a slight intent to die? If so, were these thoughts, occasional, often or very often? Were these thoughts mild, moderate or severe? Did you plan or intend to hurt yourself either actively or passively (e.g. not avoiding a risk)? If yes, did you intend to die as a result? Did you have a method or a way to kill yourself in your mind (e.g. how)? In the past month, did you do things to get ready to kill yourself, but did not start to kill yourself? Did you start to try to kill yourself, but then stopped yourself before you hurt yourself? Did you start to try to kill yourself, but then someone or something stopped you before you hurt yourself? Did you injure yourself on purpose without intending to kill yourself? Did you hope to be rescued/survive? In your lifetime, did you ever feel so bad that you wished you were dead or felt like killing yourself?

In your lifetime, did you ever do things to prepare or to get ready to kill yourself? Did you ever try to kill yourself? How many times have you ever tried to kill yourself? Which method(s) have you ever used when you tried to kill yourself?

#### Suicidality

A binary outcome variable (suicidality) was developed; if the adolescent had deliberately attempted to injure him/herself with the intention of suicide in the past month and or in their life time.

#### Socio economic status

The socio economic status was measured using an asset index created by combining data on nine [9] household items using principle component analysis.

#### HIV stigma

The Brief HIV stigma scale by Berger and colleagues was used to assess the HIV felt stigma among the participants [21]. The BHSS is a 9-item scale rated on a five-point Likert scale from “Strongly Disagree” to “Strongly Agree”.

#### Assessment of psychiatric disorders

Psychiatric disorders were assessed using the child and adolescent symptom inventory (CASI-5). It is a behaviour rating scale for emotional and behavioral disorders in children and adolescents aged 5 and 18 years old [22]. It is designed according to the fifth edition of the Diagnostic and statistical manual of mental disorders (DSM V). The CASI-5 provides an algorithm that we used to generate the different psychiatric presentations. There is a parent (173 items, 8 pages) and teacher (125 items, 7 pages) version of the CASI-5. The CASI-5 is a revised version of the Child and Adolescent Symptom Inventory-4R (CASI-4R), which combined the items from the Child Symptom Inventory-4 (CSI-4) and the Adolescent Symptom Inventory-4 (ASI-4) into a single measure.

### Procedure

The 392 study participants were identified by the research assistants using the patient register of the baseline CHAKA study. Those who met eligibility criteria were asked for consent and assent to participate in the study. This process was followed at each of the study sites until the required sample was attained. The study tools were administered by trained research assistants supervised by a clinical psychologist and a psychiatrist. The study tools were translated in Luganda (commonly spoken language in the study area) and locally adapted as described in a paper by Mpango and colleagues [23]. This aimed to make the tools understandable to the respondents. The interviews were conducted in both English and or Luganda.

### Statistical analyses

**Fig. 1:**
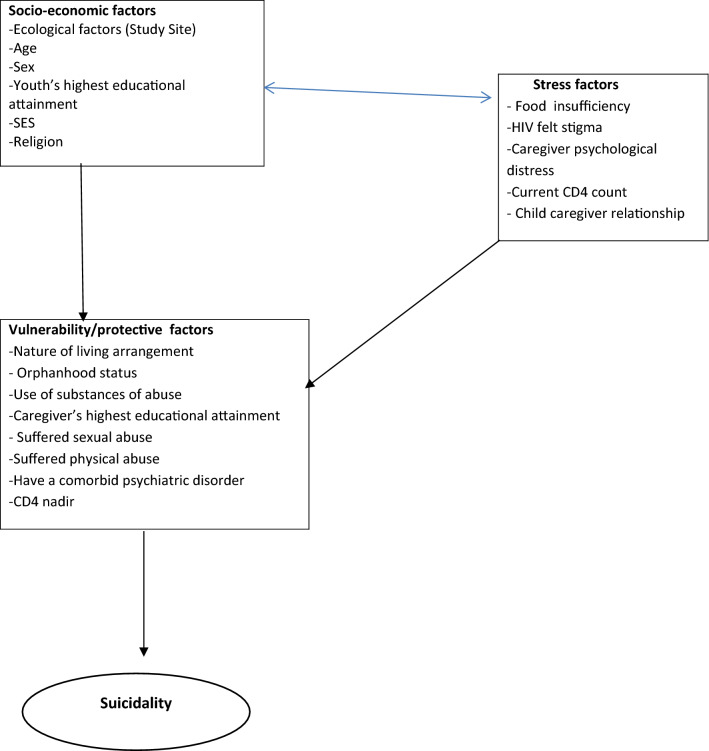
A Modified Stress-Diathesis Model guiding data analysis (Mann et al., 1999)

Socio-demographic characteristics, Psychiatric diagnoses, psychosocial and clinical factors were described. A conceptual framework (Fig. 1) based on the stress-vulnerability model for suicide was specified a priori to guide the statistical analyses of correlates of suicidality [24]. Chi square tests were initially run to find association of exposure variables with suicidality. Logistic regression was used to calculate adjusted odds ratios (aORs) adjusting for study site and sex and 95% confidence intervals (CIs) for the association with suicidality and other demographic and psychosocial factors. Factors that were found to have a *p *< 0.15 in the univariable analysis were included in an initial multivariable logistic regression model. Factors that had a p < 0.1 were retained in the final analysis. Statistical analyses were conducted using Stata 15 (Stata Corp, TX, USA). There were no corrections to alpha as this study was exploratory.

## Results

### Prevalence of suicidality

- Past month: Among the 392 youths in the study, 10.7% (n = 42, CI 8 – 14.1) had moderate to severe risk of suicidality in the past one month with higher rates among urban female youths (12.4%, CI the youths themselves, the reported life time prevalence of attempted suicide was 2.8% (n = 11, CI 1.6–5.0 (see Table 1). 8.6–17.7) than males (8.7%, CI 5.4 –13.8).

**Table 1:** Prevalence of suicidality, frequency and methods used among adolescents with HIV in Uganda (CHAKA study)

	Total (N = 392)	Gender		Locality	
		Male	Female	Urban	Rural
Caregiver reported					
Moderate to severe risk of suicidality^a^	42(10.7%) (CI 8–14.1)	16(8.7%) (CI 5.4–13.8)	26(12.4%) (CI 8.6 –17.7)	25(12.9%) (CI 8.9–18.5)	17(8.5%) (CI 5.4–13.3)
Attempted suicide lifetime^b^	9 (2.30%) (CI 1.2–4.4)	2(1.2%) (CI 0.3–4.5)	7(3.4%) (CI 1.6–7.1)	9(4.9%) (CI 2.5–9.2)	0
Youth Self-report					
Attempted suicide last month^c^	7(1.8%) (CI 0.8–3.7)	1(0.6%) (CI 0.07–3.8)	6(2.9%) (CI 1.2–6.3)	5(2.3%) (CI 1.1–6.1)	2(1.0%) (CI 0.2– 4.0)
Attempted suicide lifetime^d^	11(2.8%) (CI 1.6–5.0)	4(2.2%) (CI 0.8–5.7)	7(3.4%) (CI 1.6–6.9)	9(4.7%) (CI 2.4–8.7)	2(1.0%) (CI 0.2–3.9)
Frequency of attempted suicide (N = 11)					
1 time	6 (54.5%)	4(100%)	2(28.6%)	4(44.4%)	2(100)
2 times	3 (27.3%)	0	3(42.9%)	3(33.3%)	0
6 times	1 (9.1%)	0	1(14.3%)	1(11.1%)	0
9 times	1 (9.1%)	0	1(14.3%)	1(11.1%)	0
Methods of attempted suicide (N = 11)					
Cutting	2(18.2%)	2(50%)	0	2(22.2%)	
Use of HIV medications	2(18.2%)	1(25%)	1(14.3%	2(22.2)	0
Others(organophosphates, hanging, stabbing, self-starvation)	6(54.5%)	0	6(85.7%)	5(22.2)	1(11.1%)

Among children (5–11yrs), the caregiver reported 10/689(1.5%) had ever attempted suicide in their lifetime

^a^Moderate to severe risk of suicidality as defined by the suicidality module of the of Mini-International Neuropsychiatric Interview (M.I.N.I.)

^b^Assessed by asking the caregiver the question, ‘In the lifetime of this child has he/she ever deliberately injured him/herself?’

^c^Assessed by asking the adolescent the question, ‘In the past month, did you attempt suicide? Try to kill yourself?’

^d^Assessed by asking the adolescent the question, ‘In your lifetime, did you ever try to kill yourself?’
- Lifetime: The caregivers reported a lifetime suicidal attempt prevalence of 2.3% (n= 9, CI 1.2–4.4) among youths. According to reports by the youths themselves, the reported life time prevalence of attempted suicide was 2.8% (n=11, CI 1.6–5.0 (see Table 1).

### Methods of attempted suicide

The most common methods used during the suicide attempts were cutting with suicide intentions (18.5%) and taking overdose of HIV medications (18.5%). Among children (5–11yrs), the caregivers reported 10/689 (1.5%) had ever attempted suicide in their lifetime (see Tables 1 and 2).

**Table 2:** Socio-demographic correlates of moderate to severe suicidality as defined by the MINI

Variable	(n = 392) n (%)	Crude odds ratio (95% CI)	*P* value	Adjusted odds ratio (95% CI)	P-value
Psychosocial vulnerability/protective factors					
Sex					
Male	16/183(8.7%)	1	P = 0.238	1	P = 0.172
Female	26/209(12.4%)	1.5(0.77–2.86)		1.6(0.78–2.91)	
Study site					
Rural	17/199(8.54%)	1	P = 0.158	1	P = 0.172
Urban	25/193(12.95%)	1.6(0.83–3.05)		1.6(0.84–3.10)	
Age					
Above 15	18/154(11.69%)	1.07(0.55–2.09)	P = 0.840	1.03(0.52 –2.06)	P = 0.07
Less 14	21/191(10.99%)	1		1	
Adolescent’s education				1	
Less than primary	29/289(10.03%)	1	P = 0.47	1.16(0.56–2.36)	P = 0.29
Primary and above	13/103(12.62%)	1.29(0.64–2.59)			
Caregiver Education level					
Less than primary	20/208(9.62%)	1	P = 0.351	1	P = 0.23
Primary and above	21/166(12.65%)	1.36(0.71–2.6)		1.16(0.59–2.28)	
Religion					
Christian	35/314(11.15%)	1.27(0.54–2.98)	P = 0.57	1.27(0.54–3.00)	P = 0.28
Non-Christian	7/78(8.97%)	1		1	
Socio-economic status					
High SES	10/144(6.94%)	1	P = 0.06	1	P = 0.04
Low SES	32/248(12.90%)	1.98(0.95–4.17)		2.27(1.06 –4.87)	
Tribe					
Muganda	28/280(10.00%)	0.78(0.39–1.54)	P = 0.48	0.89(0.43P = 0.301.83)	P = 0.30
Non Muganda	14/112(12.50%)	1		1	
Living arrangement					
Both parents	8/76(10.26%)	1		1	
Others	34/314(10.83%)	1.06(0.47 – 2.40)	P = 0.88	1.09(0.48 – 2.48)	P = 0.31
Orphan status					
Orphan	28/245(11.43%)	1.23(0.62 –2.41)	P = 0.56	1.27(0.64–2.50)	P = 0.26
Not orphan	14/147(9.52%)	1		1	
Substance use					
Ever used a substance	1/2(50%)	8.47(0.52 –138.27)	P = 0.16	9.03(0.–153.70)	P = 0.14
Never used any substance	41/389(10.54%)	1		1	
Suffered sexual abuse					
Yes	2/8(25.00%)	2.87(0.56 – 14.68)	P = 0.25	2.51(0.48 – 13.02)	P = 0.21
No	40/384(10.42%)	1		1	
Suffered physical abuse					
Yes	17/140(12.14%)	1.24(0.64 –2.39)	P = 0.52	1.16(0.60–2.24)	P = 0.29
No	25/250(10.00%)	1		1	

The risk factors for suicidality were low socioeconomic status (OR = 2.27, CI 1.06–4.87, *P *= *0.04*), HIV felt stigma (OR = 2.10, CI 1.04–3.00, *P *= *0.02*) and major depressive disorder (OR = 1.80, CI 0.48–2.10, *P *= *0.04*). Conversely, ADHD was protective against suicidality (OR = 0.41, CI 0.18–0.92, *P *= *0.04*). The rest of the results are in Table 3.

**Table 3:** Clinical correlates of moderate to severe suicidality as defined by the MINI

Variable	(n = 479) n (%)	Crude Odds Ratio (95% CI)	P-value	Adjusted Odds Ratio (95% CI)	P-value
Comorbid psychiatric disorders					
Major depressive disorder					
Yes	18/161(11.18%)	1.79(0.57–2.07)	P = 0.06	1.80(0.48–2.10)	P = 0.04
No	24/231(10.39%)	1		1	
Social anxiety disorder					
Yes	25/203(12.32%)	1.42(0.74–2.72)	P = 0.29	1.6(0.8–3.15)	P = 0.14
No	17/189(8.99%)				
PTSD					
Yes	16/115(13.91%)	1.56(0.80–3.03)	P = 0.19	1.37(0.70–2.73)	P = 0.23
No	26/277(9.39%)	1		1	
ADHD					
Yes	31/325(9.54%)	0.54(0.25 –1.13)	P = 0.097	0.41(0.18 –0.92)	P = 0.04
No	11/67(16.42%)	1		1	
Oppositional defiant disorder					
Yes	35/296(11.82%)	1.7(0.73–3.97)	P = 0.19	1.65(0.70–3.89)	P = 0.17
No	7/96(7.26%)	1		1	
Conduct disorder					
Yes	22/158(13.92%)	1.73(0.91–3.29)	P = 0.09	1.60(0.81–3.14)	P = 0.14
No	20/234(8.55%)	1		1	
Caregiver psychological distress					
No	16/133(12.03%)	1		1	
Yes	26/259(10.04%)	0.82(0.42–1.58)	P = 0.55	0.75(0.38–1.47)	P = 0.24
HIV illness factors					
Current CD4 count					
<= 500	16/127(12.60%)	1.35(0.69–2.63)	P = 0.38	1.39(0.71–2.71)	P = 0.22
>500	25/259(9.65%)	1		1	
Lowest CD4 count					
<= 400	18/167(10.78%)	1.04(0.53–2.04)	P = 0.89	1.03(0.52–2.04)	P = 0.58
>400	20/193(10.36%)	1		1	
HIV felt stigma					
Yes	12/79(15.19%)	2.03(1.03–3.01)	P = 0.03	2.10(1.04–3.00)	P = 0.02
No	30/313(9.6%)	1		1	
Child caregiver relationship					
Poor	16/132(12.12%)	1.24(0.64–2.41)	P = 0.52	1.22(0.62–2.39)	P = 0.27
Good	26/260(10.00%)	1		1	
Childhood trauma					
Yes	17/140(12.14%)	1.24(0.64–2.39)	P = 0.52	1.16(0.59–2.24)	P = 0.29
No	25/250(10.00%)	1		1	
Stress factors					
Negative life events	13/97(13.40%)	P = 0.33		1.31(0.64 –2.68)	P = 0.25
Yes	29/295(9.83%)	1.4(0.71–2.85)		1	
No		1			
Family has enough food	29/309(9.39%)				
Yes	13/80(16.25%)	1	P = 0.08	1	P = 0.13
No		1.87(0.92–3.80)		1.73(0.84–3.54)	

## Discussion

The prevalence of suicidality was 10.7% and 2.8% among adolescents living with HIV and AIDS in Kampala and Masaka districts of Uganda according to caregiver and youth self-report, respectively. The youth reported life time suicide attempt prevalence of 2.8% but their caregivers reported a life time suicide attempt prevalence of 2.3% in the same youths. Among children (5–11yrs), the caregivers reported life time suicidal attempt of 1.5%. We did not ask this question of children. The clinical correlates of suicidality were low socioeconomic status; HIV felt stigma, and major depressive disorder. ADHD was protective against suicidality. The most common methods used during the suicide attempts were cutting, taking overdose of HIV medications, use of organophosphates, hanging, stabbing and self-starvation.

Many youth with suicidality will need mental health support even in their future life [10, 11]. It is also important to note that youth with the highest need for mental health care generally have least access to therapeutic support. This could be due to associated risk factors like depression that often goes undetected and untreated [3]. When psychological problems present among individuals with underlying medical conditions, the health workers tend to focus attention on the physical symptoms. The most common medical illnesses associated with psychiatric problems are those linked with much pain/suffering or stigma or disability or shame.

### Prevalence of suicidality

The caregiver reported prevalence of 10.7% among adolescents is high [3] probably due to lack of interventions targeting suicidality in this group in Uganda. With a prevalence of 10.7%, suicidality becomes more common than other health conditions like malaria, infections or other health conditions affecting the same population. The prevalence in the current study is comparable to what has been reported by other studies in Uganda and other developing countries. For example, Ashaba et al. (2018) reported a prevalence of adolescent suicidality of 13%. The population was similar but the settings were slightly different [25]. On the other hand, the prevalence in our study is lower than what has been reported in the neighbouring country of Rwanda where a prevalence of 20% was reported among HIV-positive adolescents aged 10–17 years [26]. The differences could be in the care provided and also in the different measures used in the different studies.

The adolescents reported a slightly higher rate of suicidality (2.8%) compared to that of the caregivers (2.3%). This is an indication that caregivers may not be having close relationships with adolescents. On the other hand, it could mean that adolescent do not find it easy to open up to their caregivers about their suicidality. It is also possible that the caregiver may not have sufficient time to explore the feelings of adolescents, though more investigations are required to elucidate this finding.

### Correlates

Correlates of suicidality were low socioeconomic status; HIV felt stigma and major depressive disorder. These have been reported in previous studies. In addition to HIV infection, the adolescents struggle with orphanhood and associated life challenges [8]. It is important to provide social support and improve upon SES to lower the risk for suicide among youth infected with HIV/AIDS. It was surprising to find that ADHD was protective against suicide. This is different from observations of previous studies that have reported positive association between ADHD and suicidality [27–29]. The previous studies were not among individuals with HIV. It is possible that the HIV status, antiretroviral therapy and other HIV related factors could have played role. This relationship needs more investigation in individuals living with HIV in Africa.

### Methods used for youth suicide

According to previous research, the most frequent suicide method is hanging, followed by poisoning by pesticides for females and firearms for males [30]. In our study, the most common methods used during the suicide attempts were self-cutting with suicide intentions, taking overdose of HIV medications and use of organophosphates. Overall, the patterns of suicide methods in children and adolescents reflect lethality, availability and acceptability of suicide means which happens to be country specific and age related [31].

Studies show that about 90% of teens who kill themselves have some type of mental health problem, such as depression, anxiety, drug or alcohol abuse, or a behavior problem [32, 33]. We do not report associated mental disorders in this paper.

Use of antiretroviral medications (ART) in HIV positive adolescents is coupled with challenges to the extent that some adolescents (with a higher risk for suicide) have been using ART to end their lives. Several factors make youths with HIV/AIDS to be more prone to commit suicide but it is important to point out that adolescents often tend to use what is near and readily available to them. As an implication, it is important to keep the ART with the caregivers or matron at school to prevent any likely negative outcomes.

## Conclusions

In summary, the 10.7% prevalence of youth suicidality in the past one month among perinatally infected youths of is quite high in the HAART era. The life time higher prevalence of suicidal attempt among urban female youths was consistent with reports from previous studies. The risk factors for suicidality were low socioeconomic status; HIV felt stigma and major depressive disorder. On the other hand, Attention Deficit Hyperactivity disorder (ADHD) was protective against suicidality. The most common methods used during suicide attempts were cutting, taking overdose of HIV medications, use of organophosphates, hanging, stabbing and self-starvation. Provision of social support and improved SES may help to lower the risk for suicide among youth living with HIV/AIDS.

## Data Availability

The datasets used and/or analysed during this study are available from the corresponding author on reasonable request.

## Abbreviations

ADHD: Attention–Deficit/Hyperactivity Disorder
AIDS: Acquired Immune Deficiency Syndrome
CHAKA: Mental health among HIV infected CHildren and Adolescents in KAmpala and Masaka, Uganda
MRC: Medical Research Council
UVRI: Uganda Virus Research Institute
